# Organisationale Gesundheitskompetenz in Krankenhäusern und Pflegeeinrichtungen – Stand und Perspektiven

**DOI:** 10.1007/s00103-025-04017-5

**Published:** 2025-02-24

**Authors:** Eva Maria Bitzer, Nicola Häberle, Katharina Rathmann, Zeynep Islertas

**Affiliations:** 1 Institut für Alltagskultur, Bewegung und Gesundheit, Public Health & Health Education, Pädagogische Hochschule, Freiburg, Kunzenweg 21, 79117 Freiburg, Deutschland; 2 Fachbereich Gesundheitswissenschaften, Public Health Zentrum Fulda (PHZF), Hochschule Fulda, Fulda, Deutschland

**Keywords:** Organisationale Gesundheitskompetenz, Krankenhäuser, Pflege, Deutschland, Instrumententwicklung, Organizational health literacy, Hospitals, Nursing home, Germany, Instrument development

## Abstract

**Hintergrund:**

Offen ist, zu welchem Grad Krankenhäuser und Einrichtungen der Altenpflege in Deutschland ihren Klient:innen gute gesundheitsbezogene Entscheidungen ermöglichen, d. h. organisationale Gesundheitskompetenz (oGK) zeigen, und welche Aspekte im Detail für Identifikation von Ansatzpunkten zur Förderung der oGK essenziell sind.

**Methodik:**

Grundlage bilden 3 unabhängig voneinander durchgeführte Forschungsprojekte: (1) Befragung von Krankenhäusern (KH) und Pflegeeinrichtungen (PE; EwiKo, 2021), (2) Befragung von KH (GK-KH, 2022) und (3) ein Konsensprozess zur Reduktion eines ausführlichen Selbstbewertungsinstrumentes (SAT-OHL-Hos v1.1; 2024). In (1) und (2) einbezogen wurden alle deutschen KH sowie die 1475 PE in Sachsen und Thüringen, eingesetzt wurde der HLHO-10 (Wertebereich 1 bis 7, hohe Werte = hohe oGK). Der Konsensprozess erfolgte im WHO-Netzwerk M‑POHL mit dem Ziel der Kürzung der 141 Indikatoren um etwa 2 Drittel unter Erhalt der Tiefe und Breite des Konzeptes in 3 Schritten: Priorisierung von Indikatoren auf nationaler Ebene, Zusammenführung nationaler Voten, Konsens in der internationalen Arbeitsgruppe.

**Ergebnisse:**

Auswertbare Fragebögen liegen von 195 PE, 62 bzw. 291 KH vor (Rücklauf: 13,2 %, 3,2 % bzw. 11 %). Die Mittelwerte der 10 Standards des HLHO-10 liegen zwischen 3,5 und 5,7. Substanzielle Unterschiede zwischen KH und PE bestehen nicht. Der Konsensprozess führte zu einem auf 54 Items reduzierten Instrument, diskutiert wurde u. a., was länderübergreifend zu erwarten ist.

**Diskussion:**

Erstmals liegen Daten zur oGK in der stationären Pflege vor, die selbsteingeschätzte oGK der befragten Einrichtungen liegt im mittleren bis guten Bereich, mit dem auf ca. ein Drittel gekürzten SAT-OHL-Hos steht ein „kurzes ausführliches“ und international vergleichend einsetzbares Instrument zur Verfügung, dessen empirische Prüfung ansteht.

## Abstract (en)

**Background:**

It is unclear to what extent hospitals and nursing homes in Germany make it easier for people to navigate, understand, and use information and services to take care of their health, that is, demonstrate organizational health literacy (oHL), and which aspects are essential for identifying approaches to promoting oHL.

**Methods:**

The basis is formed by three independently conducted research projects: (1) a survey of hospitals (HO) and nursing homes (NH) (EwiKo, 2021), (2) a survey of hospitals (GK-KH, 2022), and (3) a consensus process to reduce a detailed self-assessment tool (SAT-OHL-Hos v1.1; 2024). In (1) and (2), all German hospitals and the 1475 NH in Saxony and Thuringia were included, and the HLHO-10 (range 1 to 7, high values = high oGK) was used. The consensus process was carried out in the WHO network M‑POHL with the aim of reducing the 141 indicators by about two-thirds while maintaining the depth and breadth of the concept in three steps: prioritization of indicators at the national level, merging of national votes, consensus in the international working group.

**Results:**

Evaluable questionnaires were received from 195 NH, 62 HO, and 291 HO (response rate: 13.2%, 3.2%, and 11%, respectively). The mean values of the 10 HLHO-10 standards ranged from 3.5 to 5.7, with no substantial differences between hospitals and nursing homes. The consensus process led to a reduced instrument of 54 items. Among other things, the discussion addressed the question of what can be expected across countries.

**Discussion:**

For the first time, data on oHL in nursing homes are available. The self-assessed oHL of the surveyed institutions is in the medium to good range. The SAT-OHL-Hos V2, now shortened by about one-third, is a “short, detailed” and internationally applicable instrument ready for empirical testing.

## Hintergrund

Organisationale Gesundheitskompetenz (oGK) bezeichnet den Grad, zu dem Einrichtungen in der Lage sind, ihren Klient:innen, unabhängig von deren individueller Gesundheitskompetenz (GK), gute gesundheitsbezogene Entscheidungen zu ermöglichen [1–3]. Organisationen, v. a. der gesundheitlichen Versorgung, zeigen oGK, indem sie Strukturen und Prozesse so gestalten, dass Individuen, die mit und innerhalb der Organisation interagieren, Zugang zu Gesundheitsinformationen und -dienstleistungen erhalten und befähigt werden, diese zu nutzen und informierte Entscheidungen für sich und andere zu treffen [4, 5]. Indem oGK die Bedingungen adressiert, unter denen Bürger:innen Informationen einholen und Entscheidungen treffen können, nimmt sie die Organisationen in die Verantwortung, für partizipative, individuelle Entscheidungen „zu sorgen“, und erweitert die häufig auf individuelle GK eingeengte Debatte um die Orientierung an den Verhältnissen [6, 7]. Schnittstellen und -mengen zeigt oGK zu anderen Konzepten wie der Ottawa Charta (Gesundheitsförderung in Lebenswelten; [8]), zur Responsivität gesundheitlicher Versorgung [9, 10], der Patientenzentrierung [11, 12], Konzepten der Organisationsentwicklung und des Qualitätsmanagements [13, 14] sowie der betrieblichen Gesundheitsförderung [15]. Ähnlich wie diese Strategien zielt oGK darauf ab, Einzelne durch eine zielgruppenadäquate Informationsbereitstellung, niederschwellige Navigationsmöglichkeiten und Kompetenzstärkung zu befähigen, ihre Gesundheit und den Genesungsprozess aktiv mitzugestalten, und zwar nicht nur als Wert an sich, sondern auch mit dem Ziel, Patientensicherheit und Mitarbeiterorientierung zu gewährleisten, die Qualität der Versorgung zu erhalten bzw. zu verbessern und ggf. auch zur Effizienzsteigerung der Organisation und des Gesundheitssystems beizutragen [16].

Maßgeblich handlungsleitend für die Ausgestaltung, Differenzierung, Operationalisierung und Erfassung der oGK im internationalen und nationalen Diskurs sind die von Brach et al. [1] veröffentlichten „Ten Attributes of Health Literate Health Care Organizations“ [1, 17, 18]. Zwischenzeitlich sind mehr als 15 Instrumente auffindbar, unter denen der Fragebogen zu „Health Literate Health Care Organisations“ (HLHO-10; [19]), der sich mit seinen 10-Items eng an die 10 Attribute von [1] anlehnt, eines der kürzesten Instrumente ist. Entwickelt als (kurzes) Selbstbewertungsinstrument, formulieren [19] 2 grundsätzliche Anwendungsbereiche für den HLHO-10: Der erste ist die Beantwortung wissenschaftlicher Fragestellungen, beispielsweise um den Stand der oGK in Einrichtungen der gesundheitlichen Versorgung und ihre Fähigkeit, auf die Belange von Patient:innen bzw. Klient:innen einzugehen, festzuhalten. Der zweite Anwendungsbereich ist praktischer Natur: Mit dem HLHO-10 können Einrichtungen der gesundheitlichen Versorgung selbst aktiv werden, gesundheitskompetenzbezogene Stärken und Schwächen in der Organisation feststellen und Ansatzpunkte für Maßnahmen identifizieren. Erfahrungen mit dem HLHO-10 liegen bislang aus dem Setting „Krankenhaus“ vor, sowohl aus Befragungen von Leitungspersonen [20–22] als auch der Belegschaft [23, 24]. Berichtet werden Unterschiede im Ausmaß der oGK in Abhängigkeit von der Trägerschaft des Krankenhauses (wenn auch mit widersprüchlichen Ergebnissen; [20, 21]) und von der Berufs- bzw. Statusgruppe [21]. Auch wird ein positiver Zusammenhang zwischen oGK und Patientenzufriedenheit gefunden [20]. Evidenz zum Ausmaß der oGK in Organisationen der Altenpflege ist aktuell nicht verfügbar.

In Bezug auf den Umfang deutlich am anderen Ende des Spektrums bewegt sich das „International Self-Assessment Tool for Organizational Health Literacy of Hospitals, Version 1.1“ (SAT-OHL-Hos-v1.1) der „International Working Group Health Promoting Hospitals and Health Literate Healthcare Organizations“ [25]. Ursprünglich in Österreich (und in deutscher Sprache) entwickelt [26] umfasst es 141 Hauptindikatoren und gehört damit wie auch 2 weitere im deutschen Sprachraum entwickelte Selbstbewertungsinstrumente [27, 28] zu den ausführlichen Fragebögen. Inhaltlich haben diese ausführlichen Instrumente 4 Aspekte gemeinsam: (1) Sie weisen erkennbare Parallelen zum (Qualitäts‑)Management auf [14]. (2) Ihnen liegt, auch wenn sie sich nominell auf Organisationen der gesundheitlichen Versorgung im Allgemeinen beziehen, implizit oft die Organisation „Krankenhaus“ zugrunde. (3) Für die Bewertung der Indikatoren ist im Vergleich zu einem kurzen Fragebogen mehr Zeit erforderlich. (4) Die Rückmeldungen zu den wahrgenommenen Stärken und Schwächen der GK in der Organisation sind aber dafür detaillierter als die, die mit einem kürzeren Instrument erhalten werden, sodass zielgerichtete Maßnahmen zur Verbesserung abgeleitet werden können.

Noch ungeklärt ist, welche oGK-bezogenen Aspekte bei der Entwicklung eines Instruments zu beachten sind, das nicht nur die Beurteilung der oGK in einer Organisation ermöglicht (z. B. Klinik, Pflegeheim) und dessen Ergebnisse die klare Ableitung passender Fördermaßnahmen erlauben, sondern das auch international einsetzbar ist. Erst dann können auch detaillierte Erkenntnisse zur oGK standardisiert und (inter)national vergleichend erhoben analysiert werden und so Forschung und Praxis zu oGK befördern.

Der vorliegende Beitrag kombiniert die Ergebnisse von insgesamt 3 unabhängig voneinander durchgeführten wissenschaftlichen Forschungsprojekten, um folgende Forschungsfragen zur beantworten:

1. Welches Ausmaß an oGK zeigen Krankenhäuser und Einrichtungen der Altenpflege in Deutschland?
2. Welche Aspekte von oGK sollen in einem detaillierten, trotzdem handhabbaren und international einsetzbaren Instrument zur Erfassung von oGK beibehalten werden?

Um die erste Frage zu beantworten, stellen wir im ersten Teil des Beitrags Ergebnisse von 2 unabhängig voneinander, aber methodisch vergleichbar durchgeführten, bundesweiten standardisierten Online-Befragungen von Leitungspersonen in der stationären akut- bzw. pflegerischen Versorgung vor. Zur Beantwortung der zweiten Frage ziehen wir im zweiten Teil Ergebnisse aus dem deutschen Teilprojekt des M‑POHL-Netzwerkes zur organisationalen Gesundheitskompetenz heran [29].

## Teil 1: oGK in deutschen Krankenhäusern und Einrichtungen der Altenpflege

### Methodik

#### Studiendesign

Bei den beiden Erhebungen handelt es sich um Daten aus dem Projekt „Entwicklung der Gesundheitskompetenz in Einrichtungen der Gesundheitsversorgung (EwiKo)“ [27, 30] und dem Projekt „Gute Gesundheitsentscheidungen im Krankenhaus ermöglichen“ (GK-KH; [31]).

Im Projekt EwiKo wurde zwischen März und Juli 2021 eine Befragung von Leitungspersonen im Krankenhaus, in Einrichtungen der Pflege und in der Eingliederungshilfe durchgeführt. Kontaktiert wurden im Rahmen einer Online-Befragung insgesamt *N* = 3266 Einrichtungen, wobei sich die hier vorgestellten Ergebnisse ausschließlich auf Krankenhäuser (*N* = 1792, nach dem im deutschen Krankenhausverzeichnis 2018, § 108 SGB V) und Einrichtungen der Pflege (in Sachsen und Thüringen: *N* = 1474) konzentrieren. Zur Teilnahme eingeladen wurden Personen, die mittlere oder hohe Leitungsfunktionen in den Organisationen einnehmen, z. B. Einrichtungsleitung, Oberärztin/Oberarzt. Sie wurden in Anlehnung an die „Total Design Method“ [32] 2‑mal an die Teilnahme erinnert, ein Incentive gab es nicht.

Bei dem GK-KH-Projekt handelt es sich um eine zwischen November und Dezember 2022 durchgeführte, standardisierte querschnittliche Befragung, die online oder postalisch beantwortet werden konnte. Angeschrieben und zur Befragung eingeladen wurden ärztliche, pflegerische und kaufmännische Leitungen von 1250 der 1476 im deutschen Krankenhausverzeichnis gemäß § 108 SGB V gelisteten Krankenhäusern mit mehr als 50 Betten und vollständigen Kontaktangaben (Grundgesamtheit Leitungspersonen: 3301). Die Befragten wurden ebenfalls 2‑mal an die Teilnahme erinnert. Für jeden vollständig ausgefüllten Fragebogen gab es eine Spende an eine Freiburger Wohltätigkeitsorganisation.

#### Erhebungsinstrument

In beiden Studien kam der HLHO-10-Fragebogen [19] zum Einsatz. Die Antworten sind auf einer 7‑Punkte-Likert-Skala von 1 (überhaupt nicht) bis 7 (in sehr großem Maße) anzugeben. Der HLHO-10 wurde in Deutschland mit leitenden Mitarbeiter:innen zertifizierter Brustzentren validiert und weist gute psychometrische Eigenschaften auf [19, 33].

Neben dem HLHO-10 umfassten die Erhebungsinstrumente in beiden Untersuchungen Fragen zur ausfüllenden Person und zu strukturellen Merkmalen der jeweiligen Einrichtung. Die statistischen Analysen wurden mit der Software IBM® SPSS® 28.0 in einem gepoolten Datensatz durchgeführt, dabei wurden deskriptive Statistiken wie Mittelwert, Median und Prozentwerte und Konfidenzintervalle berechnet. Zur Prüfung statistisch signifikanter Unterschiede in Subgruppen (Position im Krankenhaus und unterschiedliche Untersuchungsstichproben) diente ANOVA.

### Ergebnisse

#### Stichprobenbeschreibung

Die EwiKo-Stichprobe umfasst 62 Krankenhäuser (KH) deutschlandweit (Rücklaufquote: 3,5 %) und 195 Pflegeeinrichtungen (Pflege, in Sachsen und Thüringen, Rücklaufquote: 13,2 %), die GK-KH-Stichprobe insgesamt 371 Krankenhausleitungen (Rücklaufquote: 11 %). In beiden Erhebungen sind die Einrichtungen mehrheitlich in freigemeinnütziger Trägerschaft. Je nach Datenbasis haben in den Krankenhäusern unterschiedliche Führungskräfte den Fragebogen beantwortet: In EwiKo sind unter den Teilnehmenden in Pflegeheimen rund 55 % der Personen in der Geschäftsführung bzw. kaufmännischen Leitung tätig, während in der GK-KH-Befragung in etwa die Hälfte der auswertbaren Fragebögen von Pflegedienstleitungen ausgefüllt wurden (Tab. 1).

**Tab. 1:** Trägerschaft der Einrichtung und berufliche Position der Befragten getrennt nach Art der Einrichtung und den beiden Studien („EwiKo“ und „GK-KH“)

	EwiKo				GK-KH	
	Pflege		KH		KH	
	*N*	%	*N*	%	*N*	%
	195	100	62	100	291	100
*Trägerschaft*						
Öffentlich	18	9,2	16	25,8	103	35,5
Freigemeinnützig	114	58,5	23	37,1	116	40,0
Privat	57	29,2	18	29,0	71	24,5
Andere Träger	6	3,1	5	8,1	n. e.	n. e.
*Position*						
Ärztliche Leitung^a^	59	30,3	11	17,8	90	30,9
Kaufmännische Leitung^b^	108	55,3	18	29,0	42	14,4
Pflegedienstleitung^c^	13	6,7	15	24,2	142	48,8
Andere Position	15	7,7	18	29,0	17	5,9

^a^ EwiKo: ärztliche Leitung sowie Stationsleitung; Oberarzt/Oberärztin

^b^ EwiKo: Geschäftsführung und Einrichtungsleitung

^c^ EwiKo: Pflegedienstleitung und Abteilungsleitung

*KH* Krankenhaus, *n.* *e.* nicht erhoben

#### Stand der oGK in Krankenhäusern und Pflegeeinrichtungen in Deutschland

In Tab. 2 sind für die 10 Standards des HLHO-10 die Mittelwerte, Standardabweichungen sowie 95 %-Konfidenzintervalle aus den beiden Studien getrennt für Krankenhäuser und Pflegeeinrichtungen aufgeführt.

**Tab. 2:** Deskriptive Statistik zu den HLHO-10-Items der EwiKo-Studie und der GK-KH Studie

In welchem Maße …			*N*	M	SD	95 % -KI		Min.	Max.
						UG	OG		
1.	… widmet sich die Leitung Ihres Standortes explizit dem Thema Gesundheitskompetenz (z. B. Leitbild, Personalplanung)?	Pflegeeinrichtungen EwiKo	195	5,4	1,34	5,2	5,6	1	7
		Krankenhäuser EwiKo	61	5,4	1,38	5,0	5,8	2	7
		Krankenhäuser GK-KH	371	4,7	1,61	4,5	4,9	1	7
2.	… wird das Thema Gesundheitskompetenz in Ihrem Standort in Maßnahmen des Qualitätsmanagements berücksichtigt?	Pflegeeinrichtungen EwiKo	195	5,2	1,42	5,0	5,4	1	7
		Krankenhäuser EwiKo	61	5,1	1,61	4,7	5,5	1	7
		Krankenhäuser GK-KH	371	4,4	1,62	4,3	4,6	1	7
3.	… werden Gesundheitsinformationen in Ihrem Standort unter Einbezug von Patient:innen entwickelt?	Pflegeeinrichtungen EwiKo	194	4,4	1,69	4,1	4,6	1	7
		Krankenhäuser EwiKo	62	4,3	1,85	3,8	4,7	1	7
		Krankenhäuser GK-KH	371	3,6	1,63	3,4	3,8	1	7
4.	… werden in Ihrem Standort individualisierte Gesundheitsinformationen eingesetzt (z. B. verschiedene Sprachen, Schriftgrößen, Blindenschrift)?	Pflegeeinrichtungen EwiKo	194	3,9	1,98	3,6	4,2	1	7
		Krankenhäuser EwiKo	62	3,7	1,73	3,2	4,1	1	7
		Krankenhäuser GK-KH	371	4,3	1,61	4,1	4,5	1	7
5.	… gibt es in Ihrem Standort Kommunikationsstandards, die sicherstellen, dass Patient:innen die notwendigen Informationen wirklich verstehen (z. B. Übersetzer:in, Zulassen von Denkpausen, Rückfragen einfordern)?	Pflegeeinrichtungen EwiKo	194	4,6	1,78	4,3	4,8	1	7
		Krankenhäuser EwiKo	62	4,7	1,67	4,3	5,1	1	7
		Krankenhäuser GK-KH	371	4,5	1,57	4,4	4,7	1	7
6.	… wird in Ihrem Standort Mühe darauf verwendet, dass sich Patient:innen problemlos zurechtfinden (z. B. Hinweisschilder, Auskunftspersonal)?	Pflegeeinrichtungen EwiKo	195	5,7	1,28	5,5	5,9	2	7
		Krankenhäuser EwiKo	61	5,7	1,11	5,4	6,0	2	7
		Krankenhäuser GK-KH	371	5,2	1,27	5,1	5,3	1	7
7.	… werden in Ihrem Standort Informationen für unterschiedliche Patient:innen über verschiedene Medien bereitgestellt (z. B. dreidimensionale Modelle, DVDs, Bildergeschichten)?	Pflegeeinrichtungen EwiKo	194	4,0	1,80	3,8	4,3	1	7
		Krankenhäuser EwiKo	62	3,5	1,61	3,1	3,9	1	7
		Krankenhäuser GK-KH	371	3,6	1,61	3,5	3,8	1	7
8.	… wird in Ihrem Standort sichergestellt, dass die Patient:innen besonders in kritischen Situationen (z. B. Medikamenteneinnahmen, OP-Einwilligung) wirklich alles verstanden haben?	Pflegeeinrichtungen EwiKo	194	5,1	1,37	4,9	5,3	2	7
		Krankenhäuser EwiKo	61	5,2	1,61	4,8	5,6	1	7
		Krankenhäuser GK-KH	371	5,1	1,35	5,0	5,2	1	7
9.	… kommunizieren Sie in Ihrem Standort mit dem Patienten oder der Patientin im Vorfeld offen und verständlich, welche Kosten er/sie selbst an der Behandlung zu tragen hat (z. B. Zuzahlung)?	Pflegeeinrichtungen EwiKo	195	5,7	1,33	5,5	5,9	1	7
		Krankenhäuser EwiKo	60	5,6	1,37	5,3	6,0	2	7
		Krankenhäuser GK-KH	371	5,4	1,54	5,2	5,5	1	7
10.	… werden in Ihrem Standort die Mitarbeiter:innen zum Thema Gesundheitskompetenz geschult?	Pflegeeinrichtungen EwiKo	192	4,7	1,70	4,4	4,9	1	7
		Krankenhäuser EwiKo	62	4,7	1,69	4,3	5,2	1	7
		Krankenhäuser GK-KH	371	3,8	1,66	3,7	4,0	1	7

*M* Mittelwert, *Max.* Maximum, *Min.* Minimum *OG* Obergrenze, *SD* Standardabweichung, *UG* Untergrenze, 95 %–*KI* 95 %-Konfidenzintervall

Skalenwerte liegen zwischen 1 und 7, hohe Werte repräsentieren einen hohen Umsetzungsgrad des jeweiligen Standards

Die Selbstbewertung der oGK durch die Befragten schließt Mittelwerte zwischen 3,5 (Standard 7) und 5,7 (Standard 6 und Standard 9) ein, d. h., sie schätzen die Umsetzung der Bedingungen in ihren Einrichtungen im mittleren bis guten Bereich ein. Am wenigsten umgesetzt ist nach den beiden Stichproben der EwiKo-Studie der Standard 4 (Bereitstellung individualisierter Informationen) mit mittlerem Grad der Umsetzung zwischen 3,7 und 4,3. In der GK-KH-Studie ist der Standard 3 (Entwicklung von Gesundheitsinformationen unter Einbezug von Patient:innen) am niedrigsten ausgeprägt. Neben dem Standard 9 ist der Grad der Umsetzung bei Standard 6 am höchsten, bei dem es darum geht, wie gut sich Patient:innen in der Einrichtung zurechtfinden können.

Es fällt auf, dass die beiden EwiKo-Stichproben sehr viel ähnlicher antworten als die GK-KH-Stichprobe und dass die in GK-KH Befragten die Umsetzung in den Krankenhäusern in 8 von 10 Standards, zum Teil deutlich und statistisch signifikant, weniger gut bewerten als die Krankenhäuser in der EwiKo-Studie (z. B. Standard 1 und Standard 10).

Im Vergleich zu EwiKo schätzen die Befragten der GK-KH-Studie den Umsetzungsrad in 3 Standards deutlich und statistisch hoch signifikant niedriger ein als Befragte der EwiKo-Studie. Dabei handelt es sich um Standard 1 „Fokussierung der GK durch die Leitungspersonen“ (F (2,624) = 16,35; *p* < 0,001; η^2^ = 0,05), Standard 2 „Berücksichtigung der GK im Qualitätsmanagement“ (F (2,624) = 19,16; *p* < 0,001; η^2^ = 0,06) und Standard 3 „partizipative Entwicklung von Gesundheitsinformationen“ (F (2,624) = 15,32; *p* < 0,001; η2 = 0,05).

Auch den Umsetzungsgrad der Standards 4, 6, 9 und 10 schätzen die Befragten der GK-KH-Studie, wenn auch nicht so deutlich, aber doch signifikant geringer ein als die Befragten in EwiKo: Standard 6 „navigationale GK“ (F (2,624) = 11,76; *p* < 0,001; η^2^ = 0,04), Standard 4 „Nutzung verschiedener Medien zur Information bei der Patient:innenberatung“ (F (2,624) = 4,19; *p* = 0,016; η^2^ = 0,01), Standard 9 „Kommunikation über Behandlungskosten“ (F (2,623) = 4,573; *p* = 0,011; η^2^ = 0,02) und Standard 10 „Mitarbeiter:innenschulungen“ (F (2,622) = 19,20; *p* < 0,001; η^2^ = 0,06).

Keine substanziellen Unterschiede zwischen den Einrichtungen bzw. den Befragungen bestehen bei Standard 5 „Kommunikationsstandards“. Im Vergleich zur EwiKo etwas höher schätzen die GK-KH-Befragten den Grad der Umsetzung des Standards 7 „Bereitstellung individualisierter Informationen“ ein (F (2,624) = 6,131; *p* = 0,002; η^2^ = 0,02).

#### Einschätzung der oGK nach Position der Befragten in Krankenhäusern der EwiKo- und GK-KH-Studie

Leitendes Pflegepersonal schätzt im Vergleich zu anderen Befragten das Maß der „Partizipation bei der Informationsentwicklung“ (F (3,349) = 3,79; *p* = 0,011; η^2^ = 0,032) und die „Kommunikation über Kosten in ihren Organisationen“(F (3,347) = 4,31; *p* = 0,005; η^2^ = 0,036) wie auch die „Gewährleistung des Einverständnisses der Patient:innen in Hochrisikosituationen“ (F (3,348) = 4,33; *p* = 0,005; η^2^ = 0, 36) geringer ein als Beschäftigte in anderen Positionen. Die „Schulung der Mitarbeiter:innen zur GK“ nehmen ärztliches und pflegerisches Leitungspersonal in ihrer eigenen Organisation im Vergleich zu den kaufmännischen Leitungen als weniger umgesetzt wahr (F (3,349) = 3,15; *p* = 0,025, η^2^ = 0,026).

## Teil 2: Welche oGK-Aspekte soll ein detailliertes, trotzdem handhabbares und international einsetzbares Instrument beinhalten?

Das Aktionsnetzwerk der Weltgesundheitsorganisation (WHO) „Measuring Population and Organizational Health Literacy“ (M-POHL) zielt in dem Teilprojekt „Organizational Health Literacy Hospitals“ (OHL-HOS) darauf ab, ein europaweit einsetzbares, handhabbares Instrument zu erarbeiten, um oGK international und vergleichend sichtbar zu machen, Gesundheitssysteme bzw. einzelne Sektoren miteinander zu vergleichen und voneinander lernen zu können. Als Grundlage für dieses Vorhaben dient das SAT-OHL-Hos-v1.1 [25]. Es stützt sich, wie der HLHO-10 [19] auf [1]. Dieses ursprünglich deutschsprachige [26], später ins Englische übersetzte Instrument zählt mit 8 Standards, 21 Substandards und 141 Hauptindikatoren zu den umfassendsten Instrumenten zur Erfassung von oGK. Es ist, wie andere Instrumente auch, in erster Linie als Selbstbewertungsinstrument gedacht, explizit für Krankenhäuser, wobei (noch) nicht festgelegt ist, wer die Selbstbewertung im Krankenhaus durchführen soll. Die in M‑POHL-OHL-HOS in Krankenhäusern verschiedener beteiligter Länder durchgeführte Pilotierung der Version V1.1 zeigte in erster Linie, dass das Instrument als zu umfangreich, zu lang und zu aufwendig auszufüllen erlebt wird (Ergebnisse noch nicht veröffentlicht). M‑POHL-OHL stieß daher [29] einen Kürzungsprozess an, der unter Erhalt der 8 Standards und unter gleichmäßiger Kürzung zu einem etwa auf ein Drittel reduzierten Umfang führen sollte (Tab. 3). Der Kürzungsvorgang beinhaltete 3 Arbeitsschritte und weist Ähnlichkeiten mit Vorgehensweisen anderer Arbeitsgruppen in der Entwicklung von oGK-Selbstwertungsinstrumenten (z. B. [28]) auf:

1. nationaler Austausch: Arbeitsgruppen auf nationaler Ebene waren gehalten, das gesamte Instrument zu inspizieren, die Relevanz der einzelnen Indikatoren für die Erfassung der oGK im nationalen Gesundheitssystem zu bewerten und pro Substandard Vorschläge zu machen, welche (in der Regel 2 bis 3) Indikatoren pro Substandard mit welcher Priorität beibehalten werden sollen;
2. Zusammenführung der nationalen Voten;
3. internationale Konsensustreffen im Netzwerk M‑POHL-OHL-HOS: Diskussion und Konsentierung Standard für Standard in insgesamt 9 Online-Workshops zu je 1,5 h.

**Tab. 3:** Geplantes und umgesetztes Kürzungsvorgehen des SAT-OHL-Hos-v1.1

Standard		Substandards	Indikatoren	
			Original	Nach Kürzung
1.	Best Practices für oGK in allen Strukturen und Prozessen der Organisation implementieren	3	20	9
2.	Dokumente, Materialien und Dienstleistungen unter Beteiligung von Stakeholdern entwickeln	2	7	5
3.	Die Mitarbeiter:innen fördern und für die Verbesserung der persönlichen, professionellen oGK qualifizieren	1	6	4
4.	Einfache Orientierung und leichten Zugang zu Informationsangeboten, Dokumenten und Materialien ermöglichen	4	34	15
5.	Best Practices der GK in allen Formen der Kommunikation mit Patient:innen anwenden	5	36	3
6.	Die GK von Patient:innen und Angehörigen während des Krankenhausaufenthaltes und nach der Entlassung fördern	3	20	9
7.	Die persönliche GK der Mitarbeiter:innen in Bezug auf Berufsrisiken und den persönlichen Lebensstil fördern	1	10	4
8.	Zur Förderung der GK der lokalen Bevölkerung und zur Verbreitung der organisationalen Gesundheitskompetenz in der Region beitragen	2	8	5
Gesamt		21	141	54

*GK* Gesundheitskompetenz, *oGK* organisationale Gesundheitskompetenz

Die Diskussion in den Workshops sowie die Begründung der Entscheidungen wurden in Stichworten bei jedem Item protokolliert und tabellarisch festgehalten.

Teilgenommen haben an diesem Arbeitsvorhaben M‑POHL-Mitglieder aus Österreich, Schweiz, Tschechien, Deutschland, Israel, Italien, Niederlande und Belgien.

### Ergebnisse

Mit dem gewählten Vorgehen gelang es, das Instrument von insgesamt 141 Indikatoren auf 54 zu reduzieren. Zur Illustration des Vorgehens haben wir in Tab. 4 die Indikatoren dreier Substandards zusammengestellt und berichten, wie wir als nationales Projektteam (ZI, EMB) die Indikatoren priorisiert haben und zu welchem Ergebnis die internationale Arbeitsgruppe gekommen ist.

**Tab. 4:** Nationale Prioritäten, internationaler Konsens und Diskussionspunkte am Beispiel von 3 Substandards des Self-Assessment-Tool for Organizational Health Literacy of Hospitals (SAT-OHL-Hos-v1.1)

Standard Nr.	Benennung	Nationale Priorität			Internationaler Konsens	Diskussion
		1	2	3		
*2.*	*Dokumente, Materialien und Dienstleistungen mit den Interessengruppen auf partizipative Weise entwickeln*					
*2.1*	*Das Unternehmen bezieht Patient:innen in die Entwicklung und Bewertung von patientenorientierten Dokumenten, Materialien und Dienstleistungen ein*					
2.1.1	Alle für Patient:innen relevanten Dokumente und Dienstleistungen werden gemeinsam mit Patientenvertreter:innen und Vertreter:innen von Patientengruppen entwickelt und getestet	X	–	–	X	Materialien, die nicht inhouse erstellt wurden, miteinbeziehen
2.1.2	Das Leitsystem der Organisation wird mit Patient:innen getestet und auf Basis der Ergebnisse verbessert	–	–	–	X	–
2.1.3	Leitlinien und Prozesse für das Personal zur Patientenkommunikation werden mit Vertreter:innen des Personals und der Patient:innen entwickelt und getestet	–	–	–	–	Nicht Aufgabe jedes Krankenhauses
2.1.4	(Ehemalige) Patient:innen oder ausgebildete Schauspielpatient:innen werden in die Ausbildung des Personals einbezogen, um Rückmeldungen zur mündlichen Kommunikationsfähigkeit der Mitarbeiter:innen zu geben	–	–	X	–	Nicht machbar in der Routine, eher in Universitätskliniken
2.1.5	Die Organisation implementiert ein Feedback- und Beschwerdeverfahren für Patient:innen zur Verständlichkeit von Dokumenten, Angeboten und Leistungen	–	X	–	X	Wichtiger Aspekt des Qualitätsmanagements und des Verbesserungsprozesses
*3.*	*Die Mitarbeiter:innen hinsichtlich persönlicher und organisationaler Gesundheitskompetenz fördern und schulen*					
*3.1*	*Die persönliche und organisationale Gesundheitskompetenz wird als eine wesentliche Fachkompetenz für alle in der Organisation tätigen Mitarbeiter:innen verstanden*					
3.1.1	Dokumente wie Stellenbeschreibungen, Auswahlkriterien für Bewerber:innen, Personalentwicklungspläne usw. beinhalten Gesundheitskompetenz als Kernkompetenz	X	–	–	–	–
3.1.2	Die Organisation stellt sicher, dass die Mitarbeiter:innen – insbesondere diejenigen mit Patientenkontakt und neue Mitarbeiter:innen – in Gesundheitskompetenz und patientenzentrierter Kommunikation und Informationsvermittlung geschult werden	–	X	–	X	–
3.1.3	Die Schulung der Mitarbeiter:innen zur Patientenkommunikation folgt den Prinzipen der Gesundheitskompetenz und bezieht sich auf alle Situationen, in denen Kommunikation und Informationsvermittlung eine Rolle spielen	–	–	–	–	Redundant zu 3.1.2
3.1.4	Die Mitarbeiter:innen – insbesondere diejenigen mit Patientenkontakt – erhalten regelmäßig Feedback darüber, wie gut sie kommunizieren	–	–	–	–	Nicht leistbar, wer soll das Feedback geben?
3.1.5	Interne Expert:innen für Gesundheitskompetenz dienen anderen als Vorbild, Mentor:innen und Multiplikatoren für Gesundheitskompetenz	–	–	–	–	–
*8.*	*Zur Förderung der Gesundheitskompetenz der lokalen Bevölkerung und zur Verbreitung der organisationalen Gesundheitskompetenz in der Region beitragen*					
*8.1*	*Die Organisation trägt zur Verbesserung der Gesundheitskompetenz der lokalen Bevölkerung bei*					
8.1.1	Die Organisation stellt der lokalen Bevölkerung evidenzbasierte und nichtkommerzielle Informationen über relevante Gesundheitsthemen zur Verfügung	X	–	–	X	Reichen Links zu externen Ressourcen?
8.1.2	Die Organisation fördert Initiativen zur Gesundheitsbildung und -förderung, um die Gesundheitskompetenz der lokalen Bevölkerung zu stärken	–	X	–	X	In einigen Ländern ist dies (auch/vorrangig) die Aufgabe der Primärversorgung oder von ambulanten Fachärzt:innen
8.1.3	Die Organisation führt Maßnahmen zur Verbesserung der Gesundheitskompetenz von schwer erreichbaren Patientengruppen auf lokaler Ebene durch	–	–	–	–	Das ist nicht in jedem Land umsetzbar

X: nationale Priorität: in diesem Fall die Priorität der deutschen Projektpartner für beizubehaltende Indikatoren

X: internationaler Konsens: nach Diskussion in der internationalen Arbeitsgruppe konsentiert beizubehaltende Indikatoren

Die nationalen Priorisierungsprozesse führten in den meisten Fällen nicht direkt zu einem eindeutigen Ergebnis. Ein Konsens konnte in der Regel erst nach einem intensiven Austausch von Erklärungen, Erläuterungen und Argumenten erzielt werden, von denen wir hier die 3 zentralen Diskussionslinien skizzieren:

1. Die Breite bzw. Tiefe der Indikatoren: Wie genau oder auch wie abstrakt sollen/dürfen die Indikatoren sein?
2. Was kann man von allen (!) Krankenhäusern fordern (und nicht nur von Universitätskliniken oder Lehrkrankenhäusern)?
3. Welche Funktion erfüllen Krankenhäuser im jeweiligen Gesundheitssystem? Und was kann/darf/muss man von ihnen länderübergreifend erwarten?

Ad 1: Das Ringen um einen Kompromiss zwischen Präzision und Abstraktion lässt sich an dem Substandard 3.1 „Die persönliche und organisationale Gesundheitskompetenz wird als eine wesentliche Fachkompetenz für alle in der Organisation tätigen Mitarbeiter:innen verstanden“ zeigen. Im Ausgangsformat hat der Standard 5 Indikatoren, von denen wir als nationales Team schon nur noch 2 als beizubehaltend vorgeschlagen haben (Tab. 4). In der internationalen Diskussion verständigte man sich auf den (einen) Indikator, der mit einer (möglichst präzisen) Formulierung die Qualitätsanforderung beschreibt. Darüber hinausgehende Anforderungen an die Art und Weise, wie geschult wird, ob beispielsweise in der Routineversorgung ein regelmäßiges GK-bezogenes Feedback installiert ist, GK Bestandteil von Stellenausschreibungen ist oder regelhaft bei der Auswahl von Personal berücksichtigt wird, erschienen unklar, zu kleinteilig und zu sehr in die Belange der Krankenhäuser eingreifend.

Ad 2: Die Diskussion entlang der Frage: „Was kann man von allen (!) Krankenhäusern erwarten?“, war eindrücklich beim Substandard *2.1. *„Das Unternehmen bezieht Patient:innen in die Entwicklung und Bewertung von patientenorientierten Dokumenten, Materialien und Dienstleistungen ein“. So bestand in der Abwägung schnell Einigkeit darüber, dass man nicht von allen Krankenhäusern verlangen möchte, regelhaft Schauspielpatient:innen in die Ausbildung von Gesundheitsfachkräften einzubinden oder Leitlinien und Prozesse für das Personal zur Patientenkommunikation immer zusammen mit Patient:innen zu entwickeln. Zudem wird in der überarbeiteten Form darauf hingewiesen werden, dass der Indikator 3.1. auch erfüllt ist, wenn methodisch anspruchsvoll, partizipativ *von anderen* entwickelte Dokumente und Dienstleistungen implementiert sind. Nicht verzichtet werden sollte demgegenüber auf den Indikator, der die für das institutionelle Lernen notwendige Feedback-Schleife adressiert (Tab. 4).

Ad 3: Die unterschiedlichen Aufgaben und Funktionen, die Krankenhäuser im jeweiligen nationalen Gesundheitssystem, aber auch vor dem Hintergrund unterschiedlicher regionaler und lokaler Gegebenheiten erfüllen, wurden bei dem Substandard 8.1. deutlich. In diesem Substandard geht es um die gesellschaftliche Verantwortung des Krankenhauses, die GK der lokalen Bevölkerung zu erhöhen. In der internationalen Diskussion wurde deutlich, dass diese Aufgabe in manchen Gesundheitssystemen nicht primär im Verantwortungsbereich von Krankenhäusern liegt. Die (trotzdem) beibehaltenen 2 von 3 Indikatoren sollen einen entsprechenden Kommentar erhalten und in der anstehend geplanten Pilotierung auf ihre Tauglichkeit getestet werden.

Nicht verschwiegen werden soll aber auch, dass es in der Diskussion um die Auswahl und Formulierung weniger, gleichwohl möglichst präziser Indikatoren wiederholt den Impuls gab, neue Items aufzunehmen. Diese Impulse wurden kritisch und immer vor dem Hintergrund diskutiert, das Ziel, ein auf etwa ein Drittel reduziertes Instrument, nicht zu verfehlen.

## Diskussion

Die im ersten Teil des Beitrags berichteten Ergebnisse aus den bundesweiten Befragungen liefern erste Hinweise zum Stand der Umsetzung von oGK in deutschen Krankenhäusern und Einrichtungen der Pflege. Der Grad der Umsetzung der 10 Standards variiert, liegt etwas über dem Skalenmittelpunkt und zeugt von einer durchaus kritischen Sicht der Befragten auf „ihre“ Einrichtungen. Dass der Standard „gutes Zurechtfinden im Krankenhaus“ in allen 3 untersuchten Stichproben mit am besten beurteilt wird, ist erfreulich. Nicht unerwartet sind die relativ zurückhaltenden Bewertungen rund um die Standards zu Gesundheitsinformationen (partizipative Entwicklung, Bereitstellung zielgruppenspezifischer Formate) und die Schulung der Belegschaft zum Thema Gesundheitskompetenz. Hier zeichnen sich auf Bundesebene Verbesserungsmöglichkeiten ab, die indirekt auch die Befunde aus den bevölkerungsbezogenen Studien zur individuellen Gesundheitskompetenz wiederspiegeln: Es ist in Deutschland nicht leicht, an gute gesundheitsbezogene Informationen zu gelangen [34].

Im Vergleich zu den in der Validierungsstudie befragten zertifizierten Brustzentren [19] wenden die hier befragten Krankenhäuser und Pflegeeinrichtungen seltener Kommunikationsstandards an und stellen in geringerem Ausmaß sicher, dass das Einverständnis der Patient:innen in Hochrisikosituationen vorliegt. Auch im internationalen Vergleich sind die hier vorgestellten Ergebnisse niedrig. Beispielsweise bewerten 405 Führungskräfte aus dem mittleren und höheren Management italienischer Krankenhäuser 7 der 10 Standards im Mittel zwischen 5,16 (Standard 3) und 5,97 (Standard 4) und nur bei Standard 10 ist die Bewertung schlechter als 4 (3,7; [21]). In Peking (China) bewerten Krankenhausleitungen alle 10 Attribute im Mittel mit mehr als 6 von 7 Punkten und damit noch einmal erheblich positiver als die Befragten in unseren Untersuchungen [24]. Vergleichswerte zur oGK in stationären Pflegeeinrichtungen liegen unseres Wissens bislang nicht vor. Hier schließen wir mit den vorgestellten Ergebnissen eine Lücke.

Erstaunlich sind die geringen Unterschiede in der Bewertung von Pflegeeinrichtungen und Krankenhäusern in der EwiKo-Studie. In Einrichtungen der Pflege müsste sich oGK eigentlich leichter implementieren lassen (und weiter implementiert sein) als in Krankenhäusern. Beispielsweise haben Einrichtungen der Pflege im Vergleich zu Krankenhäusern in der Regel eine einfachere Organisationsstruktur, ein weniger breites Aufgabenspektrum, längere Verweilzeiten der Bewohner:innen und damit mehr Gelegenheiten, GK zu adressieren. Ob dies tatsächlich die Umsetzbarkeit von oGK-Instrumenten und Maßnahmen erleichtert oder ob der Befund evtl. auch (nur) aus dem hohen Abstraktionsgrad des HLHO-10 resultiert, ist in künftigen Studien zu untersuchen.

Eine Schwäche der beiden Befragungen besteht in dem geringen Rücklauf, der die Übertragbarkeit der Ergebnisse einschränkt. Die EwiKo-Stichprobe ist eine willkürliche Stichprobe und hatte u. a. aufgrund der erschwerten Bedingungen während der COVID-19-Pandemie nicht den Anspruch auf Repräsentativität. Im Abgleich mit den Grunddaten der Krankenhäuser (Statistisches Bundesamt, [35]) ist auch die GK-KH-Stichprobe mit ihrem hohen Anteil an freigemeinnützigen Krankenhäusern nicht repräsentativ.

Unter der Annahme, dass sich in erster Linie schon mit Gesundheitskompetenz vertraute Einrichtungen beteiligt haben, müsste man in der Gesamtheit aller deutschen Krankenhäuser bzw. der Pflegeeinrichtungen in Thüringen und Sachsen von einem geringeren Umsetzungsgrad ausgehen. Möglicherweise ist so die im Vergleich zu EwiKo höhere Beteiligung in der GK-KH-Studie mitverantwortlich für die fast durchgängig beobachtete schlechtere Selbsteinschätzung in der GK-KH-Stichprobe. Für Unterschiede in den Befragungen ebenfalls mitverantwortlich könnten die unterschiedlichen Erhebungszeiträume sein. EwiKo fand mitten in der zweiten Welle der COVID-19-Pandemie (2021) statt, GK-KH in 2022, zu einem Zeitpunkt, an dem sich das pandemische Geschehen entspannt hatte.

Angestrebt werden sollten bei künftigen Befragungen höhere Beteiligungsraten und die Erhebung über den HLHO-10 hinausgehender Informationen, um die Aussagekraft der Selbsteinschätzungen zu erhöhen.

Der HLHO-10 stellt mit seinen 10 Standards ein kurzes Selbstbewertungsinstrument dar, dass jedoch aufgrund seines hohen Abstraktionsgrades nur bedingt konkrete Ansatzpunkte für die Weiterentwicklung in den Einrichtungen selbst liefert. Damit wurde und wird die Entwicklung umfangreicherer Instrumente begründet [28, 36, 37]. Die Erfahrungen aus der Entwicklung und Pilotierung solcher Instrumente zeigen übereinstimmend: Der Austausch über die Standards und Indikatoren mit Fachkräften in den Einrichtungen führt zu einem gemeinsamen Verständnis von Gesundheitskompetenz und entsprechend auch einem (zu Beginn in der Regel erst in Ansätzen vorhandenen) Verständnis organisationaler Gesundheitskompetenz [27, 28, 38, 39]. Allerdings ist der Aufwand, der mit der vollständigen und umfassenden Selbstbewertung einhergeht, für die Einrichtungen erheblich und resultiert in dem Wunsch nach kürzeren, aber dennoch gezielten Ansatzpunkten identifizierender Selbstbewertungstools [27]. Auch im M‑POHL-Netzwerk gab der einhellig aus den nationalen Pilotprojekten zur kulturellen Adaptation des SAT-OHL-Hos-V1.1 formulierte Wunsch nach einem kürzeren Instrument den Anstoß zu dem hier berichteten Kürzungsprozess (Ergebnisse noch nicht veröffentlicht).

Bei diesem Prozess traten zum einen Phänomene auf, wie sie auch in nationalen Entwicklungen berichtet worden sind (z. B. das Abwägen zwischen Genauigkeit und Abstraktion; [18, 28]). Zum anderen wurde deutlicher als im nationalen Kontext, dass bei der Entwicklung eines international anwendbaren Selbstbewertungsinstrumentes Bedingungen außerhalb der Organisation, z. B. Unterschiede in den Gesundheitssystemen, mitberücksichtigt werden müssen.

Mit einem Umfang von etwa einem Drittel der ursprünglichen Indikatoren (*n* = 54) gehört das Instrument nun zu den „kurzen ausführlichen“ Instrumenten (vgl. 160 Indikatoren in [26] und 155 [37]; 88–92 [16]; 87 für das Krankenhaus, 85 für die Pflege, 77–82 für die Eingliederungshilfe, 20 in leichter Sprache [27] und 77 [28]). Es ist dabei nach Einschätzung der M‑POHL-OHL-HOS-Arbeitsgruppe gelungen, die Breite und Tiefe des Konzeptes der oGK trotz der Kürzungen zu erhalten und weiterhin für das Konzept oGK zentrale Aspekte abzubilden (z. B. die nachhaltige Implementierung effektiver Kommunikationspraktiken und den Aufbau von Fähigkeiten in der Belegschaft, gesundheitskompetente Entscheidungen ihrer Patient:innen zu ermöglichen). Limitierend ist festzuhalten, dass eine empirische Prüfung des gekürzten SAT-OHL-Hos V2 in Bezug auf Akzeptanz und Praktikabilität noch aussteht und auch (noch) keine Informationen zu den psychometrischen Eigenschaften der Standards und Indikatoren vorliegen, ähnlich wie auch bei den anderen „kurzen ausführlichen“ Selbstbewertungsinstrumenten. Diese Fragen müssen in weiteren Studien beantwortet werden.

Erwähnt werden soll, dass mittlerweile auch für Deutschland Interventionen vorliegen, die Maßnahmen zur Stärkung der GK und oGK in Einrichtungen der Gesundheitsversorgung und anderen Settings adressieren. So richtet sich der „Düsseldorfer Kompass“ an die Belegschaft von gesetzlichen Krankenkassen [40] und „EwiKo“ adressiert nicht nur Krankenhäuser und Pflegeeinrichtungen, sondern auch Einrichtungen der Eingliederungshilfe [27, 30]. Auf Organisationen in verschiedenen Settings ausgerichtet ist zudem das Konzept der organisationsbezogenen Gesundheitskompetenz in urbanen Organisationen [41]. Speziell für die Alten- und Langzeitpflege entwickelt ist „QUALIPEP“ [42].

## Fazit

Krankenhäuser und Einrichtungen der Pflege schätzen ihre organisationale Gesundheitskompetenz mittelmäßig bis gut ein, am zurückhaltendsten sind sie in Bezug auf die Bereitstellung von zielgruppenspezifischen, qualitativ hochwertigen Gesundheitsinformationen und die Schulung der Belegschaft zur Gesundheitskompetenz. Ausführliche Selbstbewertungen sind erforderlich, um den Einrichtungen konkrete Ansatzpunkte für Organisationsentwicklung zu liefern. Mit dem gekürzten SAT-OHL-Hos steht nun ein „kurzes ausführliches“ und international vergleichend einsetzbares Instrument zur Verfügung, dessen empirische Prüfung ansteht.
